# Novel Bat Lyssaviruses Identified by Nationwide Passive Surveillance in Taiwan, 2018–2021

**DOI:** 10.3390/v14071562

**Published:** 2022-07-18

**Authors:** Shu-Chia Hu, Chao-Lung Hsu, Fan Lee, Yang-Chang Tu, Yen-Wen Chen, Jen-Chieh Chang, Wei-Cheng Hsu

**Affiliations:** 1 Animal Health Research Institute, New Taipei City 251203, Taiwan; schu@mail.nvri.gov.tw (S.-C.H.); fanlee@mail.nvri.gov.tw (F.L.); yctu@mail.nvri.gov.tw (Y.-C.T.); ywchen@mail.nvri.gov.tw (Y.-W.C.); jcchang@mail.nvri.gov.tw (J.-C.C.); 2 Bat Conservation Society of Taipei, Taipei City 106056, Taiwan; chaolung@batinfo.org

**Keywords:** lyssavirus, bat, Taiwan, *Nyctalus plancyi velutinus*, *Pipistrellus abramus*

## Abstract

Bat lyssaviruses were identified in Taiwan’s bat population during 2016–2017. The lyssavirus surveillance system was continuously conducted to understand the epidemiology. Through this system, the found dead bats were collected for lyssavirus detection by direct fluorescent antibody test and reverse transcription polymerase chain reaction. Three bats were identified as positive during 2018–2021. A novel lyssavirus, designated as Taiwan bat lyssavirus 2, was detected in a *Nyctalus plancyi velutinus*. This lyssavirus had less than 80% nucleotide identity in the nucleoprotein (N) gene with other lyssavirus species, forming a separate branch in the phylogenetic analysis. The other two cases were identified in *Pipistrellus abramus* (Japanese pipistrelles); they were identified to be similar to the former lyssavirus identified in 2016–2017, which was renominated as Taiwan bat lyssavirus 1 (TWBLV-1) in this study. Even though one of the TWBLV-1 isolates showed high genetic diversity in the N gene compared with other TWBLV-1 isolates, it may be a TWBLV-1 variant but not a new species based on its high amino acid identities in the nucleoprotein, same host species, and same geographic location as the other TWBLV-1.

## 1. Introduction

To date, 17 species of lyssavirus have been officially recognized by the International Committee on Taxonomy of Viruses (ICTV) [1,2,3,4]. The other two putative new lyssaviruses were published [5,6]. Over the past five years, more lyssavirus species were identified worldwide [4,5,6,7]. These novel lyssaviruses fulfilled the lyssavirus species demarcation criteria of the ICTV [8]: having less than 78–80% or 80% nucleotide identities on whole nucleoprotein (N) gene or concatenated coding regions compared to known lyssaviruses, respectively, and forming a monophyletic group in phylogenetic analysis. Although lyssaviruses are genetically various, all lyssaviruses cause the fatal disease rabies [9]. Among the lyssaviruses, they are divided into at least three phylogroups by their immunogenic properties and genetic diversity [2,10]. Current rabies vaccines confer partial or complete protection against phylogroup I lyssaviruses, but do not provide efficacious protection against lyssaviruses of phylogroups II and III [10,11,12,13,14].

Most studies currently indicate that bats are the reservoir host of lyssaviruses, except for Mokola lyssavirus (MOKV) and Ikoma lyssavirus (IKOV) [1,2,15,16]. In Asia, rabies lyssavirus (RABV) circulates in dogs and many wildlife [17,18]; in comparison, the other five lyssaviruses, Aravan lyssavirus (ARAV) [19], Khujand lyssavirus (KHUV) [19], Irkut lyssavirus (IRKV) [20], Gannoruwa bat lyssavirus (GBLV) [7], and Taiwan bat lyssavirus (TWBLV) [4], were merely found in bats. All circulating lyssaviruses in Asia belong to phylogroup I. There were two lyssaviruses discovered in Taiwan. The rabies lyssavirus was identified in *Melogale moschata subaurantiaca* (Formosan ferret-badger) in 2013 [21,22] and the other, Taiwan bat lyssavirus, was identified in *Pipistrellus abramus* (Japanese pipistrelle) in 2016 [4]. To monitor the activities of lyssavirus in Taiwan’s bat populations, a passive lyssavirus surveillance system has been continuously implemented in Taiwan for years. Thus, another novel bat lyssavirus was discovered and characterized between 2018 and 2021.

## 2. Materials and Methods

### 2.1. Sample Collection, Lyssavirus Diagnosis, and Virus Isolation

The lyssavirus surveillance system and the diagnostic methods performed in this study were described previously [23]. Briefly, the found dead bats were collected by nongovernmental organizations and local animal disease inspection authorities and were submitted to the Animal Health Research Institute in 2018–2021. The bats were necropsied in a biosafety level 2 laboratory, and their brain tissues were assayed by direct fluorescent antibody test (FAT) and reverse transcription polymerase chain reaction (RT-PCR).

Detecting lyssavirus antigen using the FAT, the brain smear was stained with each of the two commercially available FITC-conjugated anti-rabies antibodies (Catalog No. 800-092, Fujirebio Diagnostic Inc., Malvern, PA, USA; Catalog No. 5100, EMD Millipore Corporation, Temecula, CA, USA).

To test the viral RNA by RT-PCR, the brain tissue was homogenized into 10% (*w*/*v*) brain homogenate in minimum essential medium, and the supernatant was used for nucleic acid extraction after centrifugation. Detecting lyssavirus RNA by the RT-PCR, two sets of RT-PCR primers, JW12/N165-146 [24] and N113F/N304R [25,26], were used. The reagent preparation and thermal cycling conditions were followed as per the corresponding references.

Virus isolation was performed when a positive result was given by either the FAT or the RT-PCR [23]. Briefly, the supernatant of 10% (*w*/*v*) brain homogenate was mixed with a suspension of 3 × 10^6^ mouse neuroblastoma cell/mL, and the mixture was incubated at 37 °C in a 1% CO_2_ atmosphere for 1 h. After incubation, the mixed brain homogenate cell suspension was then transferred to a flask and a few control slides and cultivated for three to four days. One of the control slides was fixed and stained with FITC-conjugated anti-rabies antibodies each day for examining the possible fluorescence. Several blind passages were performed when no fluorescence was observed after the four-day incubation.

### 2.2. Identification of Bat Species

Bat species were identified by external morphological characteristics as previously described [27,28]. For the lyssavirus positive case, the bat’s species was further confirmed through the DNA barcoding [29,30,31].

### 2.3. Complete Genome Sequencing of the Lyssavirus Isolate

For the whole genome amplification of lyssavirus, twelve sets of RT-PCR primers were used [4], and a few of the primers were modified according to the sequences of the isolate (Table S1). The RT-PCRs were performed using the SuperScript III One-Step RT-PCR System with Platinum Taq polymerase High Fidelity kit (Invitrogen, Life Technologies, Carlsbad, CA, USA) following the manufacturer’s instruction. The reaction was initiated at 42 °C for 40 min and then at 95 °C for 2 min, followed by 35 cycles of 95 °C for 40 s, 50 °C for 50 s, and 72 °C for 80 s, and the reaction ended after a final extension of 72 °C for 7 min.

The termini sequences at the 3′ and 5′ ends of viral genome were obtained using the SMARTer RACE kit (Clontech Laboratories, TaKaRa Bio Company, Mountain View, CA, USA) following the manufacturer’s instructions. Due to the fact that the amount of bat brain tissue was limited, the nucleic acid extracted from the cell culture supernatant of the virus isolation was used for the amplification of the termini sequences.

The RT-PCR products were sequenced with the 3700XL DNA analyzer (Applied Biosystems, Waltham, MA, USA) by a commercial sequencing service (Mission Biotech Co., Taipei, Taiwan).

### 2.4. Phylogenetic Analysis

The obtained sequences of the N gene in this study were aligned with those of representative lyssavirus strains retrieved from GenBank using the MUSCLE program implemented in the software Molecular Evolutionary Genetic Analysis version X [32]. The best-fit model of nucleotide substitution and partition scheme was evaluated using ModelFinder implemented in the phylogenomic inference software IQ-TREE [33,34,35] The maximum likelihood phylogenetic tree was constructed with IQ-TREE2 v2.1.3 [36], and the branch supports were estimated using 1000 ultrafast bootstrap replicates [37]. The phylogenetic tree produced by the IQ-TREE was further edited and visualized by the Figtree v1.4.4 program (http://tree.bio.ed.ac.uk/software/figtree/ accessed on 16 September 2019).

The genome sequences of all representative lyssaviruses used in the analyses were Rabies lyssavirus (RABV; GenBank accession number: NC001542), Lagos bat lyssavirus (LBV; NC020807), Mokola lyssavirus (MOKV; NC006429), Duvenhage lyssavirus (DUVV; NC020810), European bat lyssavirus 1 (EBLV-1; NC009527), European bat lyssavirus 2 (EBLV-2; NC009528), Australian bat lyssavirus (ABLV; NC003243), Aravan lyssavirus (ARAV; NC020808), Khujand lyssavirus (KHUV; NC025385), Irkut lyssavirus (IRKV; NC020809), West Caucasian bat lyssavirus (WCBV; NC025377), Shimoni bat lyssavirus (SHIBV; NC025365), Ikoma lyssavirus (IKOV; NC018629), Bokeloh bat lyssavirus (BBLV; NC025251), Lleida bat lyssavirus (LLEBV; NC031955), Gannoruwa bat lyssavirus (GBLV; NC031988), Taiwan bat lyssavirus (TWBLV; NC055474), Kotalahti bat lyssavirus (KBLV; LR994545), and Matlo bat lyssavirus (MBLV; MW653808). In addition to the aforementioned representative sequences, more lyssavirus sequences of each species in GenBank were employed, and a total of 233 sequences were used in this study (Table S2). The phylogroup III lyssaviruses were used as an outgroup in the phylogenetic tree.

Furthermore, the sequences of the corresponding coding regions (i.e., N gene, phosphoprotein gene, matrix protein gene, glycoprotein gene, and RNA-dependent RNA polymerase gene) of the identified lyssavirus and the representative sequences were aligned respectively, as described before. The percentage of nucleotide identities in each gene between the identified lyssavirus and the representative sequences were calculated using the MegAlign program of the Lasergene software version 7 (DNASTAR Inc., Madison, WI, USA).

The nucleotide and amino acid sequences of the N gene of ABLV from GenBank (Table S2) were used to verify the range of the intra-genotypic nucleotide and amino acid identities of lyssavirus. The sequences were aligned as described before and the identities between nucleotide sequences and between amino acid sequences were calculated using the Sequence Identity Matrix tool in BioEdit [38] to obtain the intra-genotypic identities of the analyzed lyssaviruses.

### 2.5. Histopathological Examination

When a lyssavirus-positive case was diagnosed, its tissues of central nerve system and salivary glands were investigated for the possible lesions. These fresh tissues were fixed in 10% buffered formalin, embedded in paraffin wax, and stained with hematoxylin and eosin for histopathological examination.

## 3. Results

### 3.1. Lyssavirus Surveillance

From 2018 to 2021, 407 bat specimens were received and tested by the Animal Health Research Institute. These specimens covered at least 13 bat species and came from 18 cities or counties in Taiwan (Table S3). Of the received specimens, 62.7% (N = 225) were *Pipistrellus abramus*, which is a bat species distributing throughout Island of Taiwan (Figure S1). Three of the 407 bat specimens were positive for lyssavirus, detected by RT-PCR and FAT. Confirming the bats’ species by DNA barcoding, two in 2018 and 2020 were *P. abramus*, and the other was identified in 2018 as *Nyctalus plancyi velutinus*, which was the first case in this species found infected in Taiwan. The lyssaviruses of the three positive cases were recovered by virus isolation and named as TWBLV-1/YiL/2018, TWBLV-1/KL/2020, and TWBLV-2/NT/2018, respectively. The results of the FAT, RT-PCR, and virus isolation of the positive cases were listed in Table S4. The isolates, bat species, location, and year of the bat lyssavirus found in Taiwan were listed in Table 1.

### 3.2. Complete Genome Sequencing and Phylogenetic Analysis

**Figure 1:**
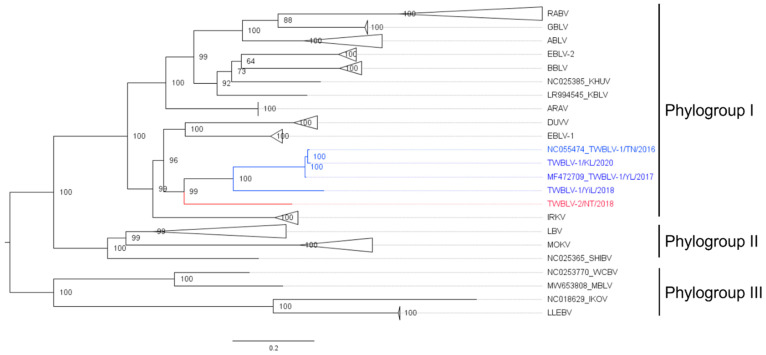
Phylogenetic relationship of a novel lyssavirus (red font) identified in *Nyctalus plancyi velutinus* in 2018, Taiwan. The nucleotide sequences of representatives of all lyssavirus species and their isolates from GenBank were analyzed. The Taiwan bat lyssavirus 1 isolates discovered in Taiwan were marked in blue font. The maximum likelihood phylogeny was constructed with full-length nucleoprotein sequences by IQ-TREE using the best fit model of nucleotide substitution with 1000 ultrafast bootstrap. Numbers at the nodes indicate ultrafast bootstrap supports. The phylogroup III lyssavirus were used as the outgroup. A clade composed of the same lyssavirus species was collapsed, and the species names are labeled on the right. The scale bar indicates the number of substitutions per site. The detailed phylogenetic tree with accession numbers of all genomes is shown in Figure S2. ABLV, Australia bat lyssavirus; ARAV, Aravan lyssavirus; BBLV, Bokeloh bat lyssavirus; DUVV, Duvenhage lyssavirus; EBLV-1, European bat lyssavirus 1; EBLV-2, European bat lyssavirus 2; IKOV, Ikoma lyssavirus; IRKV, Irkut lyssavirus; KHUV, Khujand lyssavirus; KBLV, Kotalahti bat lyssavirus; LBV, Lagos bat lyssavirus; LLEBV, Lleida bat lyssavirus; MBLV, Matlo Bat Lyssavirus; MOKV, Mokola lyssavirus; SHIBV, Shimoni bat lyssavirus; RABV, Rabies lyssavirus; TWBLV-1, Taiwan bat lyssavirus 1; TWBLV-2, Taiwan bat lyssavirus 2; WCBV, West Caucasian bat lyssavirus.

The length of the complete genome of the isolate TWBLV-2/NT/2018 was 11,990 nucleotides with the 43.17% G + C content. The nucleotide similarity between TWBLV-2/NT/2018 and the other lyssaviruses ranged 70.7–79.6% in the N gene and 63.3–76.2% in the concatenated coding genes (Table 2). Among the lyssaviruses, the N gene of TWBLV-2/NT/2018 shared the highest nucleotide identity with those of TWBLV (79.1~79.6%), EBLV-1 (79.1%), IRKV (78.1%), and DUVV (78%). The phylogenetic analysis demonstrated that TWBLV-2/NT/2018 was grouped into phylogroup I and most closely related to, but separate from, TWBLV (Figure 1). Following the species criteria of lyssavirus by the ICTV, TWBLV-2/NT/2018 was suggested to be a novel species of lyssavirus, designated as Taiwan bat lyssavirus 2 (TWBLV-2). As a new lyssavirus species discovered in a bat in Taiwan, the original TWBLV, which was identified in *P. abramus*, was renominated as Taiwan bat lyssavirus 1 (TWBLV-1) in this article.

The complete genome sequences of the isolates TWBLV-1/YiL/2018 and TWBLV-1/KL/2020 were compared to the TWBLV-1 isolates in 2016 and 2017. The nucleotide similarity between TWBLV-1/KL/2020 and TWBLV-1 identified in 2016 and 2017 was 98.5~99.0% in the N gene and 98.6~98.9% in the concatenated coding genes (Table 3). The genomic organization of the identified lyssavirus in this study are listed in Table S5.

The nucleotide similarities between TWBLV-1/YiL/2018 and the other three TWBLV-1 isolates were 81.2~81.4% in the N gene and 79.7~79.8% in the concatenated coding genes, which fulfilled the criteria in 2018 (80–82% for the complete N gene or 80% for the concatenated coding regions). Therefore, the TWBLV-1/YiL/2018 could be a novel lyssavirus. In order to verify the range of intra-genotypic nucleotide and amino acid identities of the lyssavirus, the ABLV sequences were collected from GenBank, and further analysis was performed in this study. The intra-genotypic nucleotide and amino acid identities of the N gene were 83.6~100% and 95.5~100% among ABLV (Table 3), respectively. It was assumed that the isolate TWBLV-1/YiL/2018 belonged to TWBLV-1, and the intra-genotypic nucleotide and amino acid identities of the N gene among TWBLV-1 were 81.2~99% and 96.2~100%, which was consistent with those of RABV (93.7~99.0%), EBLV-1 (97.8–100%), EBLV-2 (97.3–99.8%), and ABLV (95.5~100%) [39].

### 3.3. Histopathological Examination

**Figure 2:**
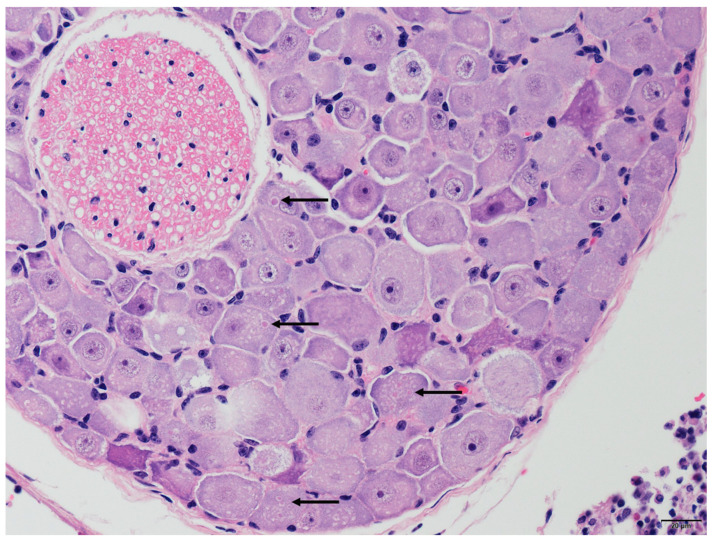
Various sized, ovoid, intracytoplasmic eosinophilic inclusion bodies (Negri bodies, represented by the arrows) were noted in the neurons of the spinal ganglion infected by the Taiwan bat lyssavirus 1 isolate TWBLV-1/KL/2020.

Two of the three positive cases, TWBLV-1/KL/2020 and TWBLV-2/NT/2018, were suitable for histopathological examination. The rest case was ruled out due to the fact of its severe degrees of postmortem changes of specimen. Neuronal degeneration and necrosis and perivascular cuffing with various numbers of mononuclear cells were observed in the cerebrum of *N. velutinus* from which TWBLV-2/NT/2018 was isolated. The pathogonomonic Negri bodies (ovoid, approximately 2 µm in length) were noted in the cytoplasm of neurons in the cerebrum of the *N. velutinus* and in the spinal ganglion of *P. abramus* from which TWBLV-1/KL/2020 was isolated (Figure 2). Lymphocytic infiltrations with varying degrees were also noted in the salivary glands in both cases.

## 4. Discussion

To the best of authors’ knowledge, we reported a novel lyssavirus, TWBLV-2, identified in *Nyctalus plancyi velutinus.* This lyssavirus had less than 80% nucleotide identity in the N gene with other lyssavirus species; thus, forming a separate branch in the phylogenetic analysis. The results revealed that the TWBLV-2 isolate in our study, TWBLV-2/NT/2018, met the new species criteria of lyssavirus by the ICTV. The phylogenetic tree based on the complete N gene showed that TWBLV-2/NT/2018 was clustered with TWBLV-1, EBLV-1, and DUVV and belonged to phylogroup I lyssaviruses.

A TWBLV-1 variant, TWBLV-1/YiL/2018, was found in a *Pipistrellus abramus* from Yilan County, Taiwan. Based on the high amino acid identity in nucleoprotein between TWBLV-1/YiL/2018 and other TWBLV-1 isolates (96.2%), in addition to the same host species (*P. abramus*) and same geographic distribution (Taiwan), we suggest that this isolate belongs to TWBLV-1 instead of a new species. Furthermore, the ICTV demarcation criteria of lyssavirus was revised down from 80–82% to 78–80% nucleotide identity for the complete N gene in 2021, thus also supporting that TWBLV-1/YiL/2018 was a variant of TWBLV-1. Unlike the variants of ABLV being identified in different bat species (*Pteropus* species and *Saccolaimus flaviventris*) [40], TWBLV-1 was identified only in *P. abramus*. Geographically, the TWBLV-1/YiL/2018 was the only TWBLV-1 isolate found in the eastern Taiwan (Figure S1). The other TWBLV-1 isolates, which were found in western Taiwan, revealed 98.5~99% nucleotide similarity in the N gene. There are mountains along central Taiwan Island from north to south. The mountainous geographic barrier with a high elevation between the eastern and western regions may contribute to the independent evolution of TWBLV-1 in *P. abramus* and, thus, results in this genetic diversity. The distinct clades of rabies lyssavirus based on geographical separation has been mentioned [41,42]; likewise, the geographical barrier causes the host genetic discontinuity [43].

In the present study, we reported that *Nyctalus plancyi velutinus* could be a potential reservoir of the TWBLV-2 by one identified case only. The more surveillance of *N. velutinus* that is performed, the more evidence will be brought up to clarify whether *N. velutinus* is a reservoir of TWBLV-2 or just incidentally infected. *N. velutinus* is the subspecies of *Nyctalus plancyi*, which is distributed in eastern China, Taiwan, and Luzon of the Philippines [44,45]. In Taiwan, *N. velutinus* is widely distributed from low to middle altitude areas; it is not a commonly observed bat species, with an unknown population size. A total number of 14 roadkill records of *N. velutinus* were received from the Taiwan Roadkill Observation Network during 2011~2021 [46]. Similarly, we received only four *N. velutinus* cases (4/407, 1%) during 2018~2021. This indicates that *N. velutinus* is a rarely observable species in the country. Even though the possibility of bat–human contact is rare, and only one TWBLV-2 isolate was found in Taiwan, our findings imply that the surrounding East Asian countries may also be exposed to a similar risk. *N. velutinus*, with similar potential long migration ability as other bats in the *Nyctalus* genus (*N. noctule* and *N. leisleri*) [47,48], may be a competent reservoir and responsible transmitter for international transmission of bat lyssaviruses.

The reservoir of TWBLV-1 was *Pipistrellus abramus* in which four cases were confirmed in Taiwan. *P. abramus* is a common insectivorous, non-migratory bat of low-altitude, urban areas in East Asia [49]. The prevalence of TWBLV-1 in *P. abramus* was (2/225, 0.89%) in 2018~2021. *P. abramus* was the bat species most commonly discovered as a victim of roadkill in Taiwan [50]. Moreover, 62.7% of received bat’s species were *P. abramus* in this study, reflecting that this species has high contact possibility with people in Taiwan. The awareness of public health should be promoted to avoid bat–human contact so as to prevent human cases.

Infection with either TWBLV-1 or TWBLV-2 in bats could develop histopathologic lesions in the central nervous system of infected bats. Even though no clear neurological symptoms were recorded in the cases, we observed characteristic Negri bodies within the neurons of the TWBLV-1 and TWBLV-2 infected bats. In the previously studies, aggression and attack behaviors were noted in experimentally inoculated bats, and some of the infected bats became emaciated and then died [51,52,53]. To further investigate clinical symptoms, the incubation period, and transmission route of Taiwan bat lyssaviruses in bats, advanced studies are required to understand the pathogenicity and pathogenesis of these bat lyssaviruses in bats.

This passive surveillance system extensively broadened our understanding of bat lyssaviruses in Taiwan. Meanwhile, it had limitations and/or bias in collecting samples of uncommon species. Though some species had few or had never been received and tested for lyssavirus in our study under the passive surveillance system, this surveillance still provided valuable information. Several surveillance systems implemented in Europe and Asia [7,20,54,55,56,57,58] indicate that passive surveillance is more suitable for identifying lyssavirus-infected bats compared to active surveillance on swabs of healthy bats [54]. Once the target of a bat species can be determined by passive surveillance, further research on pathogen monitoring, virus transmission between colonies, or seroprevalence can be conducted. Furthermore, the associated lyssavirus surveillance in other countries with the specific bat species will be encouraged to fill the gap of lyssavirus distribution in bats around the world.

In conclusion, a bat lyssavirus, designated as TWBLV-2, was newly isolated and identified in *N. velutinus*. The virus isolate fulfilled the criteria as a putatively novel species of the genus lyssavirus, and its epidemiology and pathogenicity deserve further studies.

## Figures and Tables

**Table 1:** The Lyssaviruses isolated in bats in Taiwan during passive surveillance.

Isolate Name *	Bat Species	Location	Year	GenBank Accession Number	Reference
TWBLV-1/TN/2016 †	*Pipistrellus abramus*	Tainan City, Taiwan	2016	NC055474	[4]
TWBLV-1/YL/2017 †	*Pipistrellus abramus*	Yulin County, Taiwan	2017	MF472709	[4]
TWBLV-1/YiL/2018	*Pipistrellus abramus*	Yilan County, Taiwan	2018	ON437590	This study
TWBLV-1/KL/2020	*Pipistrellus abramus*	Keelung City, Taiwan	2020	ON437591	This study
TWBLV-2/NT/2018	*Nyctalus plancyi velutinus*	New Taipei City, Taiwan	2018	ON437589	This study

* TWBLV-1: Taiwan bat lyssavirus 1; TWBLV-2: Taiwan bat lyssavirus 2. † The original name of the isolate was TWBLV (Taiwan bat lyssavirus), and TWBLV was renominated as TWBLV-1 in this study.

**Table 2:** The nucleotide identities (%) for the nucleoprotein (N), phosphoprotein (P), matrix protein (M), glycoprotein (G), RNA-dependent RNA polymerase (L) genes, and the concatenated coding genes (N + P + M + G + L) among Taiwan bat lyssavirus 2 and other lyssaviruses.

Virus Name (GenBank Accession Number)	N	P	M	G	L	Concatenated Coding Genes (N + P + M + G + L)
Rabies lyssavirus (NC001542)	74.0	62.9	72.4	62.5	71.7	70.0
Lagos bat lyssavirus (NC020807)	74.2	51.5	72.2	56.9	70.9	67.7
Mokola lyssavirus (NC006429)	73.4	51.2	70.1	56.9	70.6	67.2
Duvenhage lyssavirus (NC020810)	78.0	67.4	79.6	66.1	76.3	74.4
European bat lyssavirus 1(NC009527)	79.1	69.8	78.3	69.5	78.0	76.2
European bat lyssavirus 2(NC009528)	74.9	67.1	75.0	65.7	74.4	72.6
Australian bat lyssavirus (NC003243)	74.4	64.7	72.2	63.0	73.1	70.7
Aravan lyssavirus (NC020808)	77.7	66.2	78.0	66.5	75.2	73.6
Khujand lyssavirus (NC025385)	76.2	66.7	78.5	65.5	74.6	72.9
Irkut lyssavirus (NC020809)	78.1	69.1	78.0	66.2	75.7	74.1
West Caucasian bat lyssavirus (NC025377)	72.7	52.8	70.6	52.2	67.9	65.0
Shimoni bat lyssavirus (NC025365)	75.2	54.7	73.4	58.4	70.6	68.1
Ikoma lyssavirus (NC018629)	70.8	50.1	69.0	51.0	66.1	63.3
Bokeloh bat lyssavirus (NC025251)	74.9	67.4	76.8	64.5	74.5	72.6
Lleida bat lyssavirus (NC031955)	70.7	52.2	71.6	52.2	65.9	63.7
Gannoruwa bat lyssavirus (NC031988)	75.3	63.9	73.9	64.7	74.2	72.0
Taiwan bat lyssavirus 1 * (NC055474, MF472709)	79.1~79.6	70.3~70.6	78.7~78.8	67.9~68.6	77.2~77.4	75.6~75.7
Kotalahti bat lyssavirus (LR994545)	76.2	67.8	77.7	67.5	75.5	73.8
Matlo bat lyssavirus (MW653808)	72.3	53.5	68.8	52.2	68.7	65.4

* The original name of lyssavirus was Taiwan bat lyssavirus, and Taiwan bat lyssavirus was renominated as Taiwan bat lyssavirus 1 in this study.

**Table 3:** (A) The intra-genotypic nucleotide and amino acid identities of the nucleoprotein gene of Taiwan bat lyssavirus 1 (TWBLV-1) isolates and Australian bat lyssavirus (ABLV) isolates. (B) The percent identities of nucleotide and amino acid (underlined) between the nucleoprotein gene of Taiwan bat lyssavirus 1 (TWBLV-1) and Taiwan bat lyssavirus 2 (TWBLV-2).

(A)					
Genome	Intra-Genotypic Variation				
	TWBLV-1 *			ABLV	
	Nucleotide Identity	Amino Acid Identity		Nucleotide Identity	Amino Acid Identity
Nucleoprotein	81.2~99.0%	96.2~100%		83.6~100%	95.5~100%
**(B)**					
	**TWBLV-1/TN/2016**	**TWBLV-1/YL/2017**	**TWBLV-1/KL/2020**	**TWBLV-1/YiL/2018**	**TWBLV-2/NT/2018**
TWBLV-1/TN/2016		98.7	99	81.2	79.1
TWBLV-1/YL/2017	100		98.5	81.3	79.6
TWBLV-1/KL/2020	100	100		81.4	79.3
TWBLV-1/YiL/2018	96.2	96.2	96.2		79.6
TWBLV-2/NT/2018	93.6	93.6	93.6	93.6	

* The original name of lyssavirus was TWBLV (Taiwan bat lyssavirus), and TWBLV was renominated as TWBLV-1 in this study.

## Data Availability

All the data pertaining to the study are available in the manuscript.

## Supplementary Materials

The following are available online at https://www.mdpi.com/article/10.3390/v14071562/s1, Table S1: The modified primer used in the whole genome sequence for the isolates in this study; Table S2: The reference sequence of the lyssavirus species included in the phylogenetic tree construction; Table S3: The details of the number and bat species tested in the surveillance between 2018 and 2021; Table S4: The results of the positive cases, employed with different diagnostic methods; Table S5: The genomic organization of the identified lyssaviruses in Taiwan; Figure S1: Map of the Island of Taiwan, showing the tested number of *Pipistrellus abramus* during 2018~2021 by counties/cities; Figure S2: A detailed phylogenetic tree of the complete nucleoprotein gene of lyssaviruses.
