# Supplementary material for: Novel Bat Lyssaviruses Identified by Nationwide Passive Surveillance in Taiwan, 2018–2021
Source: Viruses. 2022 Jul 18;14(7):1562. doi: 10.3390/v14071562 (PMC9316062; doi:10.3390/v14071562)
Supplement: Supplementary file 1 [file viruses-14-01562-s001.zip › viruses-1796860-supplementary.pdf]

Supplementary File

Novel bat lyssaviruses identified by nationwide passive surveillance in Taiwan, 2018-2021

Table S1. The modified primer used in the whole genome sequence for the isolates in this study.

Primer name	Sequence, 3′---------------5′	Location (NC055474)	Isolate used
TWBLV 1F [4]	ACGCTTAACGACAAAAYC	1-18	TWBLV-2/NT/2018, TWBLV-1/YiL/2018, TWBLV-1/KL/2020
TWBLV 1R [4]	TCTTGCATTTCTTTCTCATC	1154-1173	TWBLV-2/NT/2018, TWBLV-1/YiL/2018, TWBLV-1/KL/2020
TWBLV 2F [4]	TTCGTAGGATGTTACATGGG	1010-1029	TWBLV-1/KL/2020
TWBLV 2R [4]	TAAAAATATCCCAGAAGATC	2181-2200	TWBLV-1/KL/2020
TWBLV 3F [4]	AGARATAGCWCATCAGATWGC	2125-2145	TWBLV-2/NT/2018, TWBLV-1/YiL/2018, TWBLV-1/KL/2020
TWBLV 3R [4]	CTATTGTGTGGCACCATWAC	3210-3229	TWBLV-2/NT/2018, TWBLV-1/YiL/2018, TWBLV-1/KL/2020
TWBLV 4F [4]	GATGAGGATAAGAACACATC	3078-3097	TWBLV-1/KL/2020
TWBLV 4R [4]	TCCTGAAGTGACTGAGTTTTC	4278-4298	TWBLV-1/KL/2020
TWBLV 5F [4]	CTGATGGAYGGRTCATGGGT	4085-4104	TWBLV-2/NT/2018,TWBLV-1/KL/2020
TWBLV 5R [4]	GAGACAGGAGCCGGAGTCTT	5283-5302	TWBLV-1/KL/2020
TWBLV 6F [4]	AACAGGTAGCTCCCGAGTTTGTTC	4884-4907	TWBLV-2/NT/2018, TWBLV-1/KL/2020
TWBLV 6R [4]	CTGAGTGAGACCCATGTATCCAAA	5774-5797	TWBLV-1/KL/2020
TWBLV 7F [4]	ACTGAGGTTTATGATGACCC	5485-5504	TWBLV-2/NT/2018, TWBLV-1/YiL/2018, TWBLV-1/KL/2020
TWBLV 7R [4]	CCCCAGTGTCTATARCAWCC	6568-6587	TWBLV-2/NT/2018, TWBLV-1/KL/2020
TWBLV 8F [4]	CATTCTTTGGGGGATTTCCC	6433-6452	TWBLV-1/YiL/2018, TWBLV-1/KL/2020
TWBLV 8R [4]	GTTTGTGATTCTCTRTCWATC	7602-7622	TWBLV-1/YiL/2018, TWBLV-1/KL/2020
TWBLV 9F [4]	CATGCTGGAACGGTCAGGAYG	7529-7549	TWBLV-2/NT/2018, TWBLV-1/KL/2020
TWBLV 9R [4]	CTGAGTTAAAGAAAGATTCTT	8669-8689	TWBLV-1/KL/2020
TWBLV 10F [4]	CTCAGTGAGTTRTTYAGCTC	8554-8573	TWBLV-2/NT/2018, TWBLV-1/YiL/2018, TWBLV-1/KL/2020
TWBLV 10R [4]	CAGATAGAAGAGCCTATT	9753-9770	TWBLV-1/KL/2020
TWBLV 11F [4]	CATGATTCAGGGTAYAAYGA	9655-9674	TWBLV-2/NT/2018, TWBLV-1/YiL/2018, TWBLV-1/KL/2020

TWBLV 11R [4]	GTCTGTAACTTCTGCATCAC	10859-10878	TWBLV-2/NT/2018, TWBLV-1/YiL/2018, TWBLV-1/KL/2020
TWBLV 12F [4]	ATCTGGGAAAAGCCATCAGA	10762-10781	TWBLV-2/NT/2018, TWBLV-1/KL/2020
TWBLV 12R [4]	ACGCTTAACAAAAAAAACAA	11969-11988	TWBLV-2/NT/2018, TWBLV-1/YiL/2018, TWBLV-1/KL/2020
TWBLV-2-2F	TTTGTGGGTTGCTATATGGG	1010-1029	TWBLV-2/NT/2018
TWBLV-2-2R	GAGAAATATCCCGGAGGACC	2181-2200	TWBLV-2/NT/2018
TWBLV-2-4F	GATGAGGACAAGAACACCTC	3078-3097	TWBLV-2/NT/2018
TWBLV-2-4R	TTCTAAAATGACTGAGCCTCC	4278-4298	TWBLV-2/NT/2018
TWBLV-2-5R	GAAAGGTGAACAGTTGAAAT	4633-4652	TWBLV-2/NT/2018
TWBLV-2-6R	CTGAATGAGATCCGTGTATCCACA	5774-5797	TWBLV-2/NT/2018
TWBLV-2-8F	CATTCTTTGGGTGACTTTCC	6433-6452	TWBLV-2/NT/2018
TWBLV-2-8R	CATATAAGTCGGGCATAAG	7668-7886	TWBLV-2/NT/2018
TWBLV-2-9R	CCGAATTAAAAAAGGACTCTT	8669-8689	TWBLV-2/NT/2018
TWBLV-2-10R	CAAATGGATGAACCTATT	9753-9770	TWBLV-2/NT/2018
TWBLV-1/YiL-2F	TTTGTGGGATGTTATATGGG	1010-1029	TWBLV-1/YiL/2018
TWBLV-1/YiL-2R	GAGAAATATTCCGGAGGATC	2181-2200	TWBLV-1/YiL/2018
TWBLV-1/YiL-4F	GACGAGGACAAGAACACTTC	3078-3097	TWBLV-1/YiL/2018
TWBLV-1/YiL-4R	TCCTGAAATGGCTAAGTCTTC	4278-4298	TWBLV-1/YiL/2018
TWBLV-1/YiL-5R	GGATCAGAATTTTGGGTTCA	5283-5302	TWBLV-1/YiL/2018
TWBLV-1/YiL-6F	GACAAACAAATCTTGAGGTGGTCC	4884-4907	TWBLV-1/YiL/2018
TWBLV-1/YiL-6R	CAGAGTGGGAACCATGCATCCAGA	5774-5797	TWBLV-1/YiL/2018
TWBLV-1/YiL-7R	CCCCAATGTCTGTAACACCC	6568-6587	TWBLV-1/YiL/2018
TWBLV-1/YiL-9F	CTTGTTGGAATGGTCAAGATG	7529-7549	TWBLV-1/YiL/2018
TWBLV-1/YiL-9R	CTGAGTTGAAGAAGGATTCCT	8669-8689	TWBLV-1/YiL/2018
TWBLV-1/YiL-10R	CATATGGAGGAACCTATC	9992-10009	TWBLV-1/YiL/2018
TWBLV-1/YiL-12F	ATCTGGGAGAAACCTTCTGA	10762-10781	TWBLV-1/YiL/2018

Table S2. The reference sequence of the lyssavirus species included in the phylogenetic tree construction.

Accession number	Lyssavirus name	Abbreviation
NC020808	Aravan lyssavirus	ARAV
AB094438		
NC003243	Australian bat lyssavirus	ABLV
KJ685548		
AF081020		
KT868956		
KT868955		
KT868954		

KT868953	
MK944092	
MK944091	
MK944089	
MK492317	
MK944087	
MK492319	
MK492318	
MK492316	
MK492314	
MK492315	
MK492313	
MK492309	
MK492310	
MK492311	
MK492312	
MK944083	
MK944094	
MK944082	
MK944095	
MK944093	
MK944088	
MK944090	
MK944085	
MK944084	
AF418014	
MK944086	
AY573935	
AY573936	
AY573937	
AY573938	
AY573939	
AY573940	
AY573941	
AY573942	
AY573943	
AY573944	
AY573945	
AY573946	
AY573947	
AY573948	
AY573949	

AY573950		
AY573951		
AY573952		
AY573953		
AY573954		
AY573955		
AY573956		
AY573957		
AY573958		
AY573959		
AY573960		
AY573961		
AY573962		
AY573963		
AY573964		
AY573965		
GU992312		
NC025251	Bokeloh bat lyssavirus	BBLV
MF043188		
MF197740		
KC169985		
JF311903		
LT839644		
LT839643		
LT839617		
LT839642		
NC020810	Duvenhage lyssavirus	DUVV
KC866301		
GU992315		
EU623437		
JN986749		
EU293120		
EU293119		
EU623444		
NC009527	European bat lyssavirus 1	EBLV-1
KF042302		
KF042301		
KC567818		
KF186269		
KC567816		
KC567813		
GU992303		

EU636792		
EU636789		
MF187880		
MF187877		
MF187875		
MF187861		
MF187858		
MF187843		
MF187826		
MF187824		
MF187803		
MF187801		
MF197744		
MF187855		
MF197745		
MF187874		
MF187871		
MF187831		
MF187821		
MF187808		
KF155003		
LT839608		
EU626551		
MF187815		
MF187862		
MF187820		
MF187846		
EU293112		
MF187851		
MF187848		
MF187844		
MF187834		
MF187828		
MF187814		
MF187859		
NC009528		
GU992316		
KY688152		
KY688154	European bat lyssavirus 2	EBLV-2
KY688151		
MG760848		
KY688149		

KY688144		
KY688140		
KY688143		
KY688136		
KY688134		
KY688155		
KY688137		
KY688133		
NC031988	Gannoruwa bat lyssavirus	GBLV
KU244268		
KU244267		
KU244269		
NC018629	Ikoma lyssavirus	IKOV
NC020809	Irkut lyssavirus	IRKV
JX197457		
MF737385		
NC025385	Khujand lyssavirus	KHUV
NC020807	Lagos bat lyssavirus	LBV
KP994623		
KP994622		
MH643892		
MH643891		
MH643890		
HM179509		
EF547459		
EF547458		
EF547457		
EF547456		
EF547455		
EF547454		
EF547453		
EF547452		
EF547451		
EF547450		
EF547449		
EF547448		
EF547447		
DQ499948		
DQ499947		
DQ499946		
DQ499945		
DQ499944		

JX901139		
EU293110		
NC031955	Lleida bat lyssavirus	LLEBV
MG983927		
NC006429	Mokola lyssavirus	MOKV
KP899612		
KP899611		
KC218934		
KC218933		
KC218932		
JN944637		
GU992313		
GU992310		
FJ465417		
FJ465415		
FJ465413		
FJ465412		
KF155005		
KF155006		
KF155008		
EU293117		
EU293118		
NC001542	Rabies lyssavirus	RABV
MW690155		
KX533960		
KX447689		
KX434483		
MW055229		
MW055206		
MW055143		
MW055118		
MW055086		
MN510453		
LC550025		
MT241245		
MK124737		
MN642580		
MN384718		
MN968377		
MK511227		
MF467497		
LC455982		

MG383887		
KY124533		
MG201878		
MH258829		
MF574198		
KP881356		
KP860182		
KP860174		
KP860170		
NC025365	Shimoni bat lyssavirus	SHIBV
NC055474	Taiwan bat lyssavirus 1	TWBLV-1
MF472709		
NC025377	West Caucasian bat lyssavirus	WCBV
LR994545	Kotalahti bat lyssavirus	KBLV
MW653808	Matlo bat lyssavirus	MBLV

Table S3. The details of the number and bat species tested in the surveillance between 2018 and 2021. Lyssavirus-positive cases are indicated (in brackets).

Species	2018	2019	2020	2021	species number
Pipistrellus abramus	81 (1)	89	38 (1)	47	255 (2)
Miniopterus fuliginosus	8	5	4	6	23
Scotophilus kuhlii	2	1	11	9	23
Hipposideros armiger terasensis	2	4	6	4	16
Eptesicus serotinus horikawai	2	5	7	1	15
Pipistrellus sp.		1	8	3	12
Rhinolophus monoceros	3	3			6
Murina puta	3		3		6
Nyctalus plancyi velutinus	1 (1)			3	4 (1)
Pterpus dasymallus formosus		1			1
Rhinolophus formosae			1		1
Myotis fimbriatus taiwanensis			1		1
Myotis secundus				1	1
Kerivoula titania				1	1
unidentified species	23	7	10	2	42
Total number	125 (2)	116	89 (1)	77	407 (3)

Table S4. The results of the positive cases, employed with different diagnostic methods.

Isolate name*	Direct fluorescent antibody test		RT-PCR*		Virus isolation
	FITC-conjugated antibody (Catalog No. 5100)	FITC-conjugated antibody (Catalog No. 800-092)	JW12/N165-146	N113F/N304R	
TWBLV-1/YiL/2018	+	±	+	+	+
TWBLV-1/KL/2020	+	−	+	+	+
TWBLV-2/NT/2018	+	+	+	+	+

*: RT-PCR: reverse transcription polymerase chain reaction

Table S5. The genomic organization of the identified lyssaviruses in Taiwan.

	TWBLV-1/TN/2016<br>TWBLV-1/YL/2017	TWBLV-1/YiL/2018	TWBLV-1/KL/2020	TWBLV-2/NT/2018
3′ UTR	70	69	69	69
N protein	1356	1356	1356	1356
N-P	99	100	99	106
P protein	897	894	897	894
P-M	82	82	82	80
M protein	609	609	609	609
M-G	212	212	212	212
G protein	1629	1623	1629	1623
G-L	518	529	518	524
L protein	6384	6384	6384	6384
5′ UTR	132	131	132	133
Genome	11988	11989	11987	11990

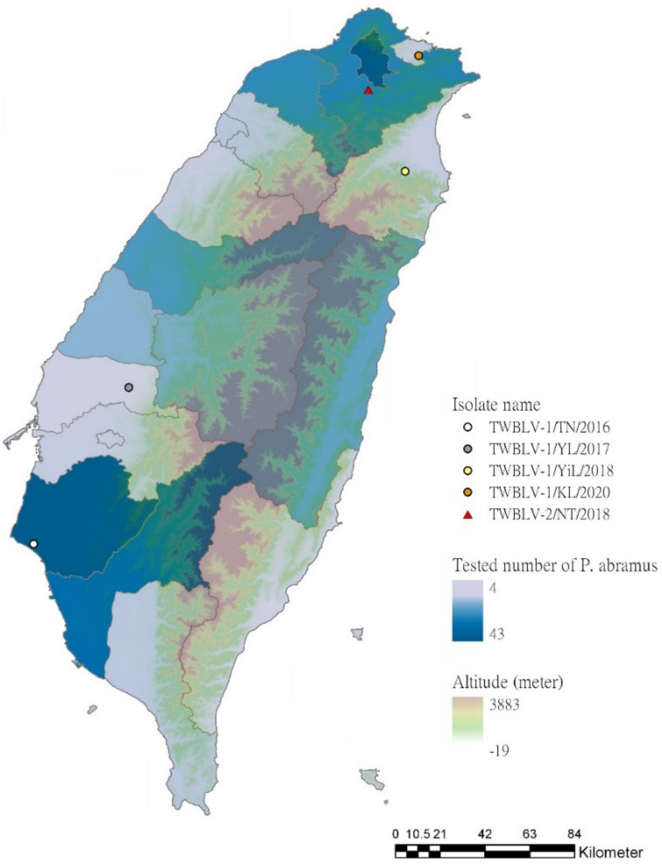

Figure S1. Map of Taiwan island, showing the tested number of *Pipistrellus abramus* during 2018~2021, by counties/cities. The location of the identified bat lyssaviruses isolates also indicated.

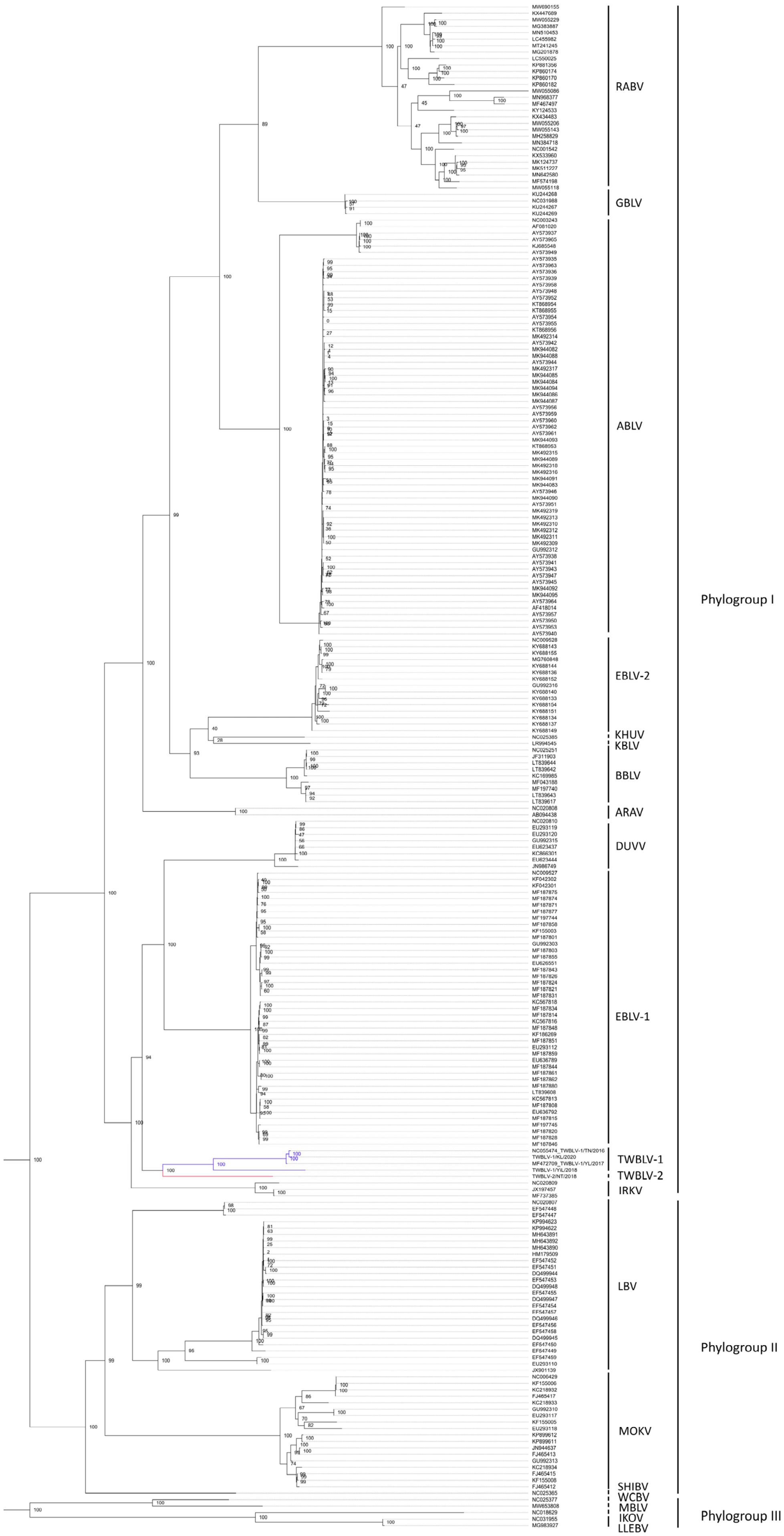

RABV
GBLV
ABLV
EBLV-2
KHUV
KBLV
BBLV
ARAV
DUVV
EBLV-1
TWBLV-1
TWBLV-2
IRKV
LBV
MOKV
SHIBV
WCBV
MBLV
IKOV
LLEBV
Phylogroup I
Phylogroup II
Phylogroup III

Figure S2. The detail phylogenetic tree of the complete nucleoprotein gene of lyssaviruses.
